# The complete chloroplast genome of *Petasites japonicus* (Siebold & Zucc.) Maxim. (Asteraceae)

**DOI:** 10.1080/23802359.2021.2005476

**Published:** 2021-11-29

**Authors:** Tamaki Hashimoto, Hiromi Tsubota, Masaki Shimamura, Yuya Inoue

**Affiliations:** a Department of Biological Science, Graduate School of Science, Hiroshima University, Hiroshima, Japan; b Miyajima Natural Botanical Garden, Graduate School of Integrated Sciences for Life, Hiroshima University, Hiroshima, Japan; c Program of Basic Biology, Graduate School of Integrated Sciences for Life, Hiroshima University, Hiroshima, Japan; d Department of Botany, National Museum of Nature and Science, Ibaraki, Japan; e Hattori Botanical Laboratory, Miyazaki, Japan

**Keywords:** Complete chloroplast genome, East Asia, Japan, long petiole, Petasites, phylogenetic relationships, Senecioneae, vegetable

## Abstract

The complete chloroplast (cp) genome sequence of *Petasites japonicus* (Asteraceae) was determined. The cp genome is 150,445 bp and consists of a large single-copy region (82,910 bp), a small single-copy region (17,907 bp), and a pair of inverted repeats (24,814 bp). It encodes a set of 114 genes, consisting of 80 protein-coding genes, 30 tRNA genes, and four rRNA genes. Phylogenetic inference confirmed that *P. japonicus* is sister to the genus *Ligularia* in the tribe Senecioneae of Asteraceae.

*Petasites japonicus* (Siebold & Zucc.) Maxim. is a rhizomatous perennial plant of the family Asteraceae, which is native to Korea, China, Japan, and Sakhalin. The species is distributed widely in Japan, ranging from Hokkaido in the north to the Ryukyu Islands in the south (Koyama 1995). After the production of inflorescences in early spring, vegetative leaves with a reniform blade and a long petiole are developed. The petioles and inflorescences are commonly used vegetables (fuki) in Japan for many centuries (Iwamoto 2009). *Petasites japonicus* shows wide morphological, ecological, and cytological variation among Japanese populations (e.g. Imazu 1961; Imazu and Fujishita 1961, 1962a, 1962b; Hashimoto et al. 2019; Shimamura et al. 2020). Plants with larger blades (reaching 1.5 m wide) and longer petioles (to 2.0 m long), distributed from Hokkaido to northern Tohoku, have been identified as a subspecies, *P. japonicus* subsp. *giganteus* (F. Schmidt ex Maxim.) Kitam. (Kitamura 1942; Koyama 1995). Genetic variations within populations and subspecies have not been evaluated, although several haplotypes have been confirmed based on the limited number of samples with nuclear internal transcribed spacers 1 and 2 including 5.8S (ITS) and chloroplast (cp) *ndhF-rpl32* and *rpl32-trnL* sequences (Steffen et al. 2016). Here we present the complete cp genome of *P. japonicus* as a resource for future genetic studies on the species. The phylogenetic relationships within the tribe Senecioneae (Asteraceae) were also inferred based on cp genome sequences with maximum likelihood analysis.

A sample of *P. japonicus* was collected from Miyagi Prefecture, northern Japan (38°54'33.9″N, 140°48'57.7″E). The specimen was deposited at the Herbarium of Hiroshima University (HIRO; Director: Tomio Yamaguchi, yamatom@hiroshima-u.ac.jp) under the voucher number HIRO-MY 140584. Fresh leaves were used for DNA extraction. Total DNA was extracted with NucleoBond HMW DNA (Macherey-Nagel, Düren) following the manufacturer’s protocols and sequenced using the Illumina MiSeq platform. A total of approximately 16 M raw reads, comprising an average fragment length of 150 bp from MiSeq. Low-quality reads (30 or less of Q-score), abnormal short reads (10 bp or less), and adapter sequences were trimmed using fastp ver. 0.20.0 (Chen et al. 2018). After quality control, the GetOrganelle ver. 1.6.4 (Jin et al. 2020) was used to assemble the filtered reads with the default seed as the probe. The assembled sequence was annotated using GeSeq ver. 1.80 (Tillich et al. 2017) and manually corrected using the SnapGene ver. 5.2.3 (from GSL Biotech; available at snapgene.com). The final annotated cp sequence was submitted to the DNA Data Bank of Japan (DDBJ) and assigned accession no. LC600309.

The complete cp genome sequence of *P. japonicus* was 150,445 bp in length; the smallest among the published cp genomes of the tribe Senecioneae (Asteraceae). The cp genome had a GC content of 37.6% and a typical quadripartite structure, consisting of a large single-copy (LSC) region of 82,910 bp, a small single-copy (SSC) region of 17,907 bp, and a pair of inverted repeats (IRs) of 24,814 bp. The cp genome contained 114 genes, including 80 protein-coding genes, of which two (*ycf1* and *rps19*) have pseudogenised copies, 30 tRNA genes, and four rRNA genes. Among them, two genes (*clpP* and *ycf3*) possess two introns, and 11 genes (*atpF*, *ndhA*, *ndhB*, *rpl2*, *rps16*, *trnA-UGC*, *trnG-UCC*, *trnI-GAU*, *trnK-UUU*, *trnL-UAA*, and *trnV-UAC*) have a single intron. The *rps12* gene was found to be trans-spliced, with one of its exons located in the LSC and the others in the IRs.

The 80 protein-coding genes of the cp genome were used to infer the phylogenetic position of *P. japonicus*. Ingroup species consisted of 22 accessions of Senecioneae, and one outgroup species, *Achyrachaena mollis* Schauer of the tribe Madieae (Asteraceae) that was selected based on the results of Gichira et al. (2019). Sequences were aligned using the program MAFFT ver. 7.475 (Katoh and Standley 2013), with some manual adjustments to the sequence editor of MEGA ver. 7.0.26 (Kumar et al. 2016). Start and stop codons were removed, and gaps were treated as missing data. Prior to the phylogenetic reconstruction, Kakusan4 (ver. 4.0.2016.11.07; Tanabe 2011) was used to determine the appropriate substitution model and partitioning scheme for our data based on the corrected Akaike information criterion (AICc: Sugiura 1978). Maximum likelihood (ML) phylogenetic analysis was performed using RAxML ver. 8.2.9 (Stamatakis 2014) with the equal mean rate model among codon positions (GTR + Γ for all regions), and a bootstrap analysis of 1,000 replicates. The resultant tree strongly supported the sister relationship of *P. japonicus* to the genus *Ligularia* Cass., which also has leaves of long petioles and reniform blades (Figure 1).

**Figure 1.**
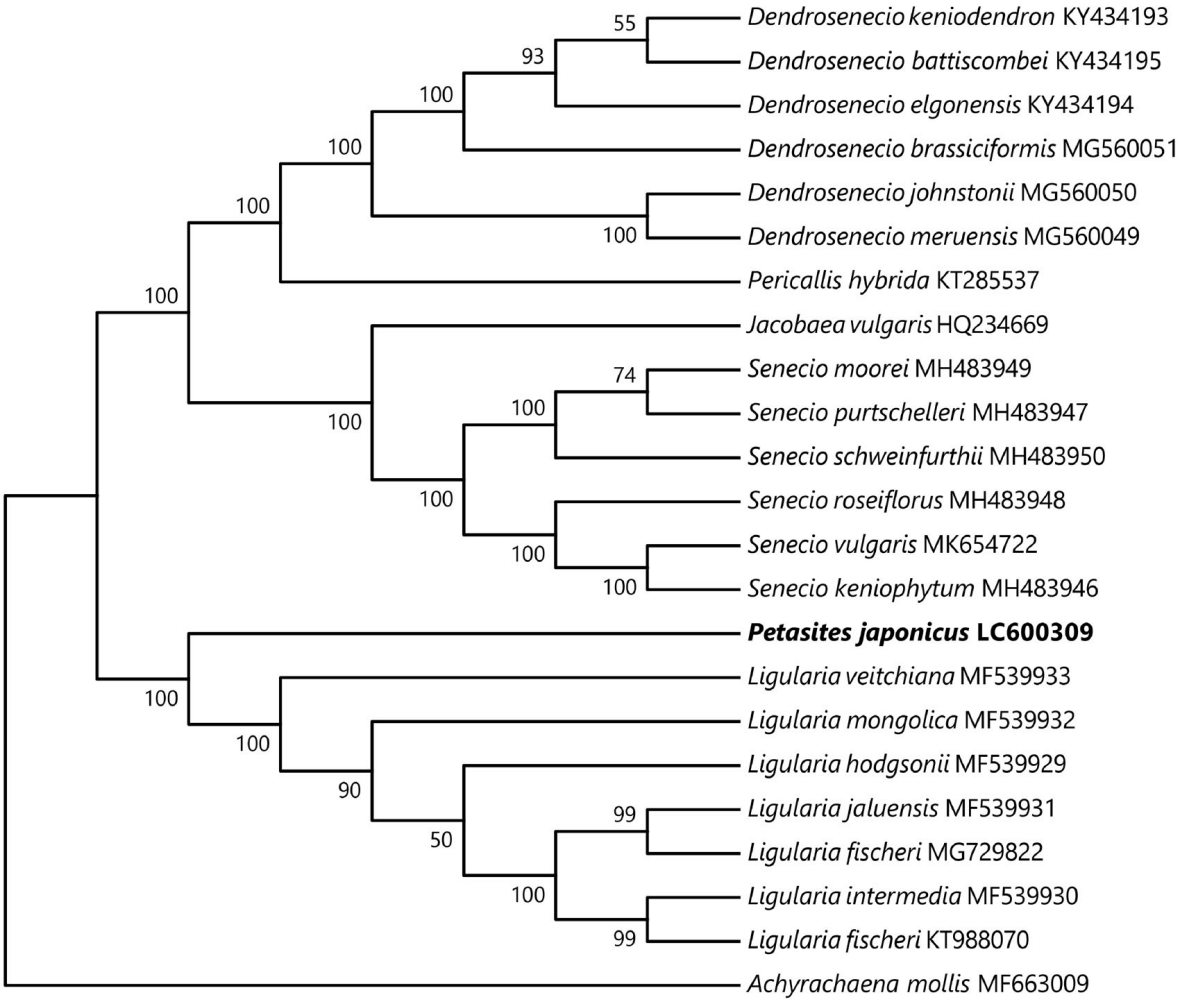
Phylogenetic relationships within 22 accessions of Senecioneae and one accession (*Achyrachaena mollis* MF663009) of Madieae as outgroup, inferred from 80 protein coding sequences with maximum likelihood method by RAxML. Bootstrap values of 1000 replicates by RAxML are shown on the branches.

## Data Availability

The genome sequence data that support the findings of this study are openly available in GenBank of NCBI at https://www.ncbi.nlm.nih.gov/ under the accession no. LC600309. The associated BioProject, BioSample, and SRA numbers are PRJDB11884, SAMD00334532, and DRR305121 respectively.
